# Border sharpness of scar tissue after myocardial infarction as determined by self-navigated free-breathing isotropic 3D whole-heart inversion recovery magnetic resonance

**DOI:** 10.1186/1532-429X-18-S1-P74

**Published:** 2016-01-27

**Authors:** Tobias Rutz, Giulia Ginami, Davide Piccini, Jérôme Chaptinel, Simone Coppo, Gabriella Vincenti, Matthias Stuber, Juerg Schwitter

**Affiliations:** 1 Division of Cardiology and Cardiac MR Center, University Hospital of Lausanne, Lausanne, Switzerland; 2 Center for Biomedical Imaging (CIBM) & Center for Cardiovascular Magnetic Resonance Research (CVMR), University of Lausanne, Lausanne, Switzerland; 3 Advanced Clinical Imaging Technology, Siemens Healthcare IM BM PI, Lausanne, Switzerland; 4 Department of Radiology, University Hospital and University of Lausanne, Lausanne, Switzerland

## Background

The border zone of myocardial scar after myocardial infarction (MI) plays an important role for arrhythmia formation. For this reason, high-resolution 3D information of scar tissue for planning of electrophysiological interventions after MI is highly desirable. This study evaluates sharpness of the borders (SB) of scar after MI by a self-navigated isotropic 3D free-breathing whole-heart magnetic resonance with inversion recovery (3DSN-IR) in comparison to a standard 2D inversion recovery sequence.

## Methods

Patients after MI detected by 2D late gadolinium enhancement (2D LGE) on a standard 2D inversion recovery sequence (resolution 1.3 mm^2^, 8 mm slice thickness) underwent 3DSN-IR on a 1.5T cardiac magnetic resonance scanner (MAGNETOM Aera, Siemens). Data acquisition was performed during the most quiescent systolic phase with a prototype 3D radial trajectory with self-navigation [1] after administration of 0.2 mmol/kg of Gadobutrol. A non-selective IR pulse was added prior to each acquired k-space segment to the segmented, ECG-triggered, fat-saturated radial SSFP imaging sequence with an isovolumetric resolution of 1.15 mm^3^. Inversion time was assessed with a 2D radial scout scan prior to 3DSN-IR. To determine SB, a customized software was used to calculate signal intensity gradients between two regions [2]. SB in mm^-1^ of borders "blood pool to scar", "blood pool to non-infarcted myocardium" and "scar to non-infarcted myocardium" were compared between a 2D LGE short-axis slice with 8 mm slice thickness and two corresponding reconstructed 3DSN-IR short-axis slices, one with isovolumetric voxel size (1.15 mm^3^) and the second interpolated to 8 mm slice thickness, all at the same anatomical location.

## Results

Thirteen patients (5 females, 58 ± 10 y, time between 2D LGE and 3D LGE 59 ± 64 days) were included. All scars visualized by 2D LGE could be identified by 3DSN-IR. SB was significantly better in 3DSN-IR compared to 2D LGE for the borders "blood pool to non-infarcted myocardium" and "scar to non-infarcted myocardium". There was a trend to a better SB for 3DSN-IR images for the border "blood pool to scar" (see table and figure).

## Conclusions

High resolution 3DSN-IR improves delineation of myocardial scar after MI as expressed by increased border sharpness in comparison to 2D LGE.

**Table 1:** Border sharpness of 2D LGE and 3DSN-IR images. Border sharpness of "blood pool to non-infarcted myocardium"," blood pool to scar" and "non-infarcted myocardium to scar" in mm-1. Data are mean ± standard deviation or range (interquartile) where appropriate.

	2D LGE	3DSN-IR isovolumetric voxel (1.15 mm)	3DSN-IR 8 mm slice thickness	p
Blood pool - non-infarcted myocardium	0.029 (0.022, 0.058)*	0.067 (0.04, 0.095)	0.071 (0.051, 0.10)	0.037
Blood pool - scar	0.083 ± 0.056	0.121 ± 0.070	0.124 ± 0.070	0.176
Scar - non-infarcted myocardium	0.079 ± 0.034†	0.171 ± 0.086	0.172 ± 0.074	<0.001

* p = 0.011 2D LGE to 3DSN-IR 8 mm slice thickness, † p < 0.006 2D LGE vs. 3DSN-IR isovolumetric voxel and 3DSN-IR 8 mm slice thickness

**Figure 1:**
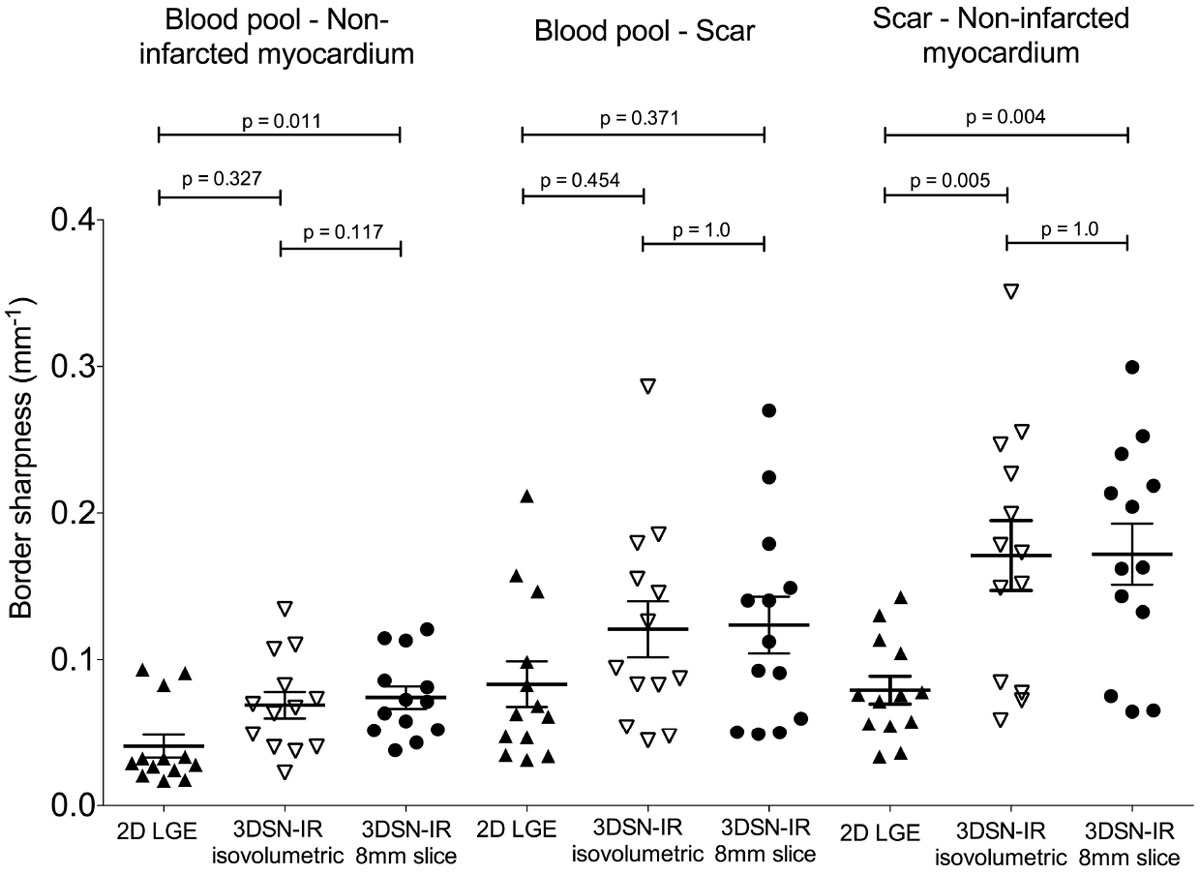
**Analyses of sharpness of the borders "blood pool to non-infarcted myocardium", "blood pool to scar" and "scar to non-infarcted myocardium" for 2D LGE short-axis slices (resolution 1.3 mm**^**2**^**, slice thickness 8 mm) and the two respective reconstructed 3DSN-IR short-axis slices, one slice with isovolumetric resolution of 1.15 mm**^**3**^ **(3DSN-IR isovolumetric) and a second slice interpolated to 8 mm slice thickness (3DSN-IR 8 mm slice)**.
